# Learning From Queues: Operational Analysis and Performance Evaluation of Dialysis Duration in a Paired Kidney Exchange With an *M/M*/1 System

**DOI:** 10.1002/lrh2.70107

**Published:** 2026-07-19

**Authors:** Samrajya Raj Acharya, Sushil Ghimire, Ram Prasad Ghimire

**Affiliations:** ^1^ Department of Mathematics, School of Science Kathmandu University Dhulikhel Nepal

**Keywords:** healthcare operations research, healthcare performance evaluation, interarrival time, learning health systems, *M/M/*1 model in healthcare, paired kidney exchange, service time, stochastic modeling in healthcare, transplant logistics

## Abstract

**Introduction:**

The allocation and timing for kidney matching and transplantation among incompatible donor‐recipient pairs in the Paired Kidney Exchange face significant operational challenges of managing an uncertain waitlist. The time for dialysis of patients waiting for biologically compatible kidneys follows the concept of a stochastic queueing system. This study embodies the Learning Health System approach by integrating real‐world data and mathematical modeling to generate actionable insights that guide decision‐making.

**Methods:**

This work employs an M/M/1 model with Poisson arrivals and exponentially distributed service times for incompatible donor–recipient pairs in a single transplant facility. Inter‐arrival and service times are used to derive queue length and waiting‐time measures to characterize system congestion and instability, with performance measures evaluated as indicative approximations analyzing the corresponding metrics.

**Results:**

The system is persistently overloaded, with arrival rates exceeding service rates (λ > μ; ρ> 1), causing prolonged waiting times. Analytical and Hutson‐B bootstrap confidence interval analysis shows congestion persists across plausible variations, and sensitivity analysis indicates moderate perturbations do not alleviate overload. Regression and correlation analyses indicate that delays are associated with arrival pressure and biologically imposed constraints, supporting compatibility‐constrained matching as the primary operational challenge. Patients experience uncertain, extended waiting periods, reflecting stochastic instability in biologically constrained queueing systems.

**Conclusions:**

Transplant service management requires systematic reforms, as clinical matching and allocation delays hinder timely care. Establishing a national transplant registry, integrated real‐time queue monitoring, decentralized planning with expansion of transplant centers and improved inter‐center coordination for paired exchange and increasing its cycle lengths are essential to ensure efficient service delivery and informed healthcare policy. These interventions create a continuous feedback loop where operational data informs queueing‐theoretic analysis, informs policy, system redesign, and ultimately improves patient access and outcomes, demonstrating how data‐driven learning optimizes complex healthcare services.

## 1 Introduction

For patients with end stage renal disease (ESRD), kidney transplantation remains the most effective treatment. However, a large proportion of patients are unable to proceed with living donor transplantation due to biological incompatibility. Paired kidney exchange (PKE) programs have emerged as a vital mechanism to overcome this issue by enabling incompatible donor–recipient pairs to exchange kidneys with other pairs [1].

**FIGURE 1:**
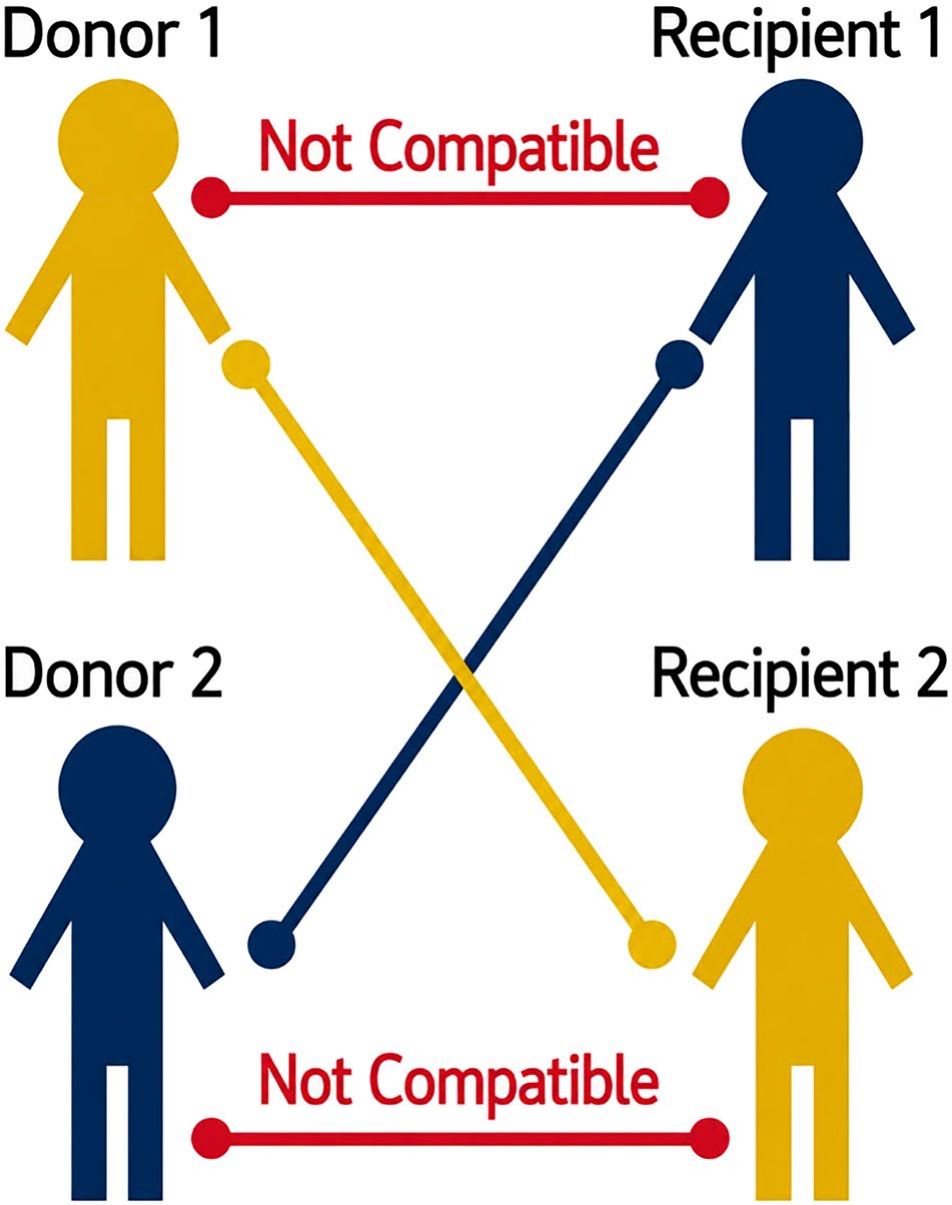
Paired kidney exchange with cycle length 2 (*Source:* UC Davis).

In Figure 1, the simplest form of PKE is examined in which incompatible donor‐recipient pairs exchange kidneys in a cycle length of 2. Here, the donor (Donor 1) associated with patient (Recipient 1) is biologically incompatible with Recipient 1 but compatible with Recipient 2, and the donor (Donor 2) associated with Recipient 2 is incompatible with Recipient 2 but compatible with Recipient 1; the two donors can exchange kidneys, forming a two‐way (pairwise) exchange. Since biological incompatibility prevents direct transplantation, a donor instead donates to a compatible recipient in another pair, provided that the original patient receives a compatible kidney in exchange. PKE arrangements are not limited to one‐to‐one exchanges as in Figure 1. While two‐way cycles are the most prevalent in practice, larger donor pools can support longer exchange cycles involving multiple donor‐recipient pairs or as chains initiated by an altruistic donor, where a non‐directed donor who is donating his organ of free will without any intended recipient can initiate the PKE. This allows the chain to continue and benefit multiple recipients even without forming a closed cycle. These structures expand matching opportunities but also increase coordination and logistic complexity. Despite their clinical and social value, PKE programs face significant challenges in managing waiting times, as patient arrivals, donor availability, and compatibility constraints introduce uncertainty into the matching process, leading to prolonged waiting times. Instead, waiting times in PKE arise from the interaction of stochastic arrivals, biological compatibility constraints, and dynamic matching decisions. The pool of incompatible donor–recipient pairs forms a compatibility graph whose structure evolves unpredictably as new pairs arrive; consequently, existing pairs depart due to transplantation or dropout, and clinical conditions change at any random point in time. Feasible exchanges depend only on the presence of compatible cycles, which may or may not exist even when surgical capacity and donors are available. As a result, patients remain in the system for prolonged periods despite adequate clinical resources, highlighting that waiting time in PKE is influenced not only by donor availability but also by mathematical and logistical considerations arising from compatibility‐constrained matching. From an operational perspective, PKE matching involves difficult intertemporal trade‐offs. Executing a feasible exchange immediately may reduce short‐term waiting times but can prevent the formation of larger or more efficient matches in the future, while waiting for better matches increases the risk of prolonged dialysis, patient dropout, or medical deterioration and even victimization. These decisions must be made under uncertainty, without full knowledge of future arrivals or compatibility patterns. Consequently, PKE systems exhibit characteristics of stochastic queueing networks with combinatorial constraints, where classical intuition such as higher arrival rates leading to longer queues or higher service capacity reducing delays does not always hold. These characteristics make rigorous operational evaluation and policy design mathematically challenging [2]. Such waiting times are associated with adverse outcomes, including prolonged dialysis, reduced quality of life, and increased morbidity risk, highlighting the urgency of developing robust approaches for system evaluation [3].

The Learning Health System (LHS) offers a useful framework for tackling these challenges. It is defined as a system where internal data and practical experience are consistently combined with external evidence, with a focus on ongoing cycles of learning and improvement [4]. Another key feature that makes LHS unique is its emphasis on continuous cycles of data‐to‐knowledge and knowledge‐to‐performance, where data from everyday practice are systematically analyzed to produce evidence that feeds back into improving care and system performance [5]. Within this setting, mathematical modeling, especially queueing theory, provides a structured way to examine waiting times in PKE programs. Queueing models capture system behavior, including uncertain patient arrivals, matching chances, and service capacity, and turn these into analytical forms that measure performance and support decision‐making. Embedding queueing analysis within the LHS allows health systems to draw insights from day‐to‐day operations, adjust allocation strategies, and strengthen system adaptability.

This study broadens the scope of LHS research by using queueing theory in the setting of PKE, where waiting time is an important yet often overlooked aspect of system performance. While many earlier works have concentrated on matching algorithms or clinical outcomes, this study's focus is on how delays build up within the PKE queue and how these delays affect system efficiency and patient access. Drawing on transplant service data, queueing‐based performance measures are utilized to quantitatively evaluate operational performance, assess potential constraints, and inform strategies for improving resource allocation and service delivery. While prior research has made substantial contributions to matching algorithms, allocation efficiency, and clinical outcomes, waiting time has often been treated as a secondary or implicit consequence rather than as a measurable and analyzable feature of system performance. This is partly because biological compatibility constraints and stochastic arrivals make delays difficult to interpret using standard operational tools. As a result, existing studies rarely provide a quantitative evaluation of the operational factors contributing to congestion within functioning PKE programs. By modeling waiting time through queueing‐based metrics grounded in real‐world data, this study treats delays as operational indicators that can be systematically evaluated to characterize congestion and support continuous monitoring within an LHS framework. Placing this analysis within the LHS cycle shows how operational data can be turned into practical insights that shape adaptive policy, reduce inefficiencies, and enhance patient outcomes. In doing so, the study fills a gap in the literature by treating waiting time not just as a clinical issue but also as a feature of healthcare delivery that can be measured and optimized within an LHS framework.

To frame the methodology, it is useful to briefly review the development of queueing theory and its applications in organ transplantation. The concept of queue has been applied in the different sectors though there are challenges to manage it efficiently. The formal study of queuing theory began in 1909 by the Danish engineer A. K. Erlang [6]. His pioneering paper in 1909 laid the groundwork for his further studies where he developed the formulae, which provided a method to calculate the likelihood of congestion rate and the probability of queue drops based on traffic load and the number of available servers. In 1953, D. G. Kendall [7] introduced a standardized notation known as Kendall's notation to classify different queuing models. More recent work has looked into variations like working vacations and threshold policies, studying how queues with fluctuating service rates during vacation periods can better represent real‐world systems with interruptions. Queueing models have been extensively used in organ transplantation, optimize transplant waiting lists, improve the efficiency of organ transplant, and implement organ allocation policies. It helps to predict waiting times, prioritize patients using the decision process, and optimize donor‐recipient matching in kidney exchanges closely echoing the principles of LHS, where models are adapted to mirror real‐life variability and drive system improvement. By enabling data‐driven prioritization and adaptive allocation, queueing models contribute to the kind of continuous system learning emphasized in LHS research.

In healthcare and organ allocation, researchers have used queueing theory to model patient‐ donor exchanges and improve transplant scheduling. Zenios et al. [8] studied a queueing model with reneging behavior of patient to represent the transplant waiting list, considering patient dropout due to death, and provided asymptotic expressions for stationary waiting times and transplantation probabilities under randomized organ allocation policies. Sekercioglu et al. [9] analyzed various methods of operations research to optimize kidney allocation highlighting their strengths and limitations to improve transplantation processes. Sowndharya et al. [10] analyzed the M/M/n queueing model to determine the optimal number of servers needed to minimize patient waiting time, improving efficiency and resource allocation. Bittencourt et al. [11] explored the application of queueing theory to optimize hospital management focusing on resource allocation, patient flow and service efficiency. Lohiya et al. [12] applied queueing theory to design and develop an optimization model to improve patient flow, resource allocation, and reducing waiting times. David et al. [13] introduced a time‐dependent stopping problem model for live organ transplant to help the decision‐ making process of accepting or rejecting organ offers. Drekic et al. [14] developed models for waiting times in deceased‐donor transplant queues including parameters like reneging patients and competing risks. Zhang et al. [15] analyzed the impact of pausing organ transplants during a global pandemic on patient survival, assessing the consequences of delayed procedures. RenDong et al. [16] introduced a hierarchy‐based M/M/n queueing algorithm to reduce waiting times for kidney transplants.

Boxma et al. [17] extended double matching queues in organ transplantation to study the impact of waiting times and allocation efficiency for both patient and donor. Chang et al. [18] explored how patient heterogeneity differences in urgency and compatibility factor affect kidney transplant waiting times under an FCFS model. Ding et al. [19] proposed a priority ranking system for cadaver kidney allocation to reduce queueing time and improve efficiency. Silva et al. [20] developed a machine learning‐based prediction model to estimate the waiting time for kidney transplantation to support transplant planning and decision‐making processes. Cechlárová et al. [21] developed a stochastic model for the kidney transplant program in Slovakia, focusing on waiting time uncertainty and probability of renal transplantations. Ratcliffe et al. [22] studied the effect of different allocation policies on the survival rate of patients in liver transplantation using mathematical simulation. Lal et al. [23] proposed a queueing‐inventory system that imitates the searching and matching process in organ allocation. Chaib et al. [24] introduced a mathematical model for pancreas‐kidney transplants to study how less‐than‐ideal inadequate donors could lower waiting time and expand access. Zenios et al. [25] pioneered dynamic allocation models that include patient‐specific traits and disease progression to optimize kidney distribution. They used clinical and statistical data to make better transplant decisions. Senanayake et al. [26] studied time‐to‐event modeling approaches to assess the cost‐effectiveness of using marginal quality kidneys, adding an economic angle to transplant decisions.

Although many studies have applied queueing theory to organ transplantation, it is observed that most existing models focus on theoretical formulations or isolated algorithmic improvements without fully capturing the day‐to‐day dynamics of a real transplant program. In turn, this poses a significant challenge for healthcare workers in fully grasping the underlying complexities required to formulate clear policies and implement its practical management. Prior research has substantially advanced matching algorithms, optimization of donor‐recipient allocation, and predictive modeling of transplant outcomes. Matching algorithms have made important progress in identifying efficient exchanges among incompatible donor–recipient pairs. However, most of these methods rely on highly idealized assumptions about donor pools and largely overlook the stochastic nature of patient arrivals and biological compatibility constraints. Queueing and simulation studies offer useful insights into waiting times and resource utilization, but they often focus on cadaveric transplantation or rely on synthetic datasets, which limits their applicability to the realities of operational PKE programs. In recent years, machine learning methods have been applied to predict patient waiting times. While these models can be effective from a predictive standpoint, they are often difficult to interpret and offer limited insight into the operational factors driving delays. As a result, their usefulness for evaluating operational performance, assessing potential constraints, and supporting adaptive management decisions remains limited. Using real transplant service data, this study treats waiting times as observable signals of system performance within an LHS framework. Building on the queueing‐based performance measures introduced earlier, this study uses real‐world data to transform waiting time into a measurable operational indicator that supports the evaluation of congestion and potential constraints, while providing a quantitative basis for continuous monitoring, data‐driven feedback, and iterative improvement of allocation strategies and program management. The arrivals are modeled as a Poisson process and the service times follow an exponential distribution in a single transplant center that is a single server. Key factors like arrival rates and service rates are characterized. Different performance measures of the M/M/1 queueing system have been calculated and numerically interpreted from the anonymized available data of the only transplant center having a PKE program in Nepal. The system has been evaluated system under a hypothetical steady‐state approximation to illustrate the magnitude of imbalance between demand and service capacity. By integrating this analysis within an LHS perspective, the study links operational modeling with real‐world data, enabling iterative learning and providing actionable guidance for policy and program management.

### 1.1 Question of Interest

How can a queueing‐theoretic framework, informed by real‐world transplant service data, be applied to evaluate the operational performance of a paired kidney exchange program, characterize patient waiting times and congestion, inform operational decision‐making, and support continuous learning within a Learning Health System?

## 2 Mathematical Model and Methodology

The dataset spans from the time patients are waiting for a paired exchange, from 2069 B.S. to 2080 B.S. Arrivals up to 2072 B.S. and from 2076 B.S. to 2077 B.S. and from 2078 B.S. to 2080 B.S. are considered as a single unit due to fewer transplants during those time periods.^1^ The period up to 2072 B.S. corresponds to the pre‐program phase, prior to the formal initiation of the national PKE program following the legalization of PKE in Nepal through the 2072 B.S. amendment of the *Organ Transplant Act of Nepal* (*2055*). Although a single patient entered dialysis in 2069 B.S. while awaiting a compatible donor, there were no active PKE queue entries in 2070 B.S. and 2071 B.S. Consequently, the pre‐2072 B.S. period was aggregated and treated as a single phase, with 2072 B.S. marking the first year of formal matching and queue activity. Similarly, the periods 2076–2077 B.S. and 2078–2080 B.S. coincide with the COVID‐19 pandemic, during which nationwide lockdowns and healthcare disruptions substantially constrained transplant activity. These years recorded fewer than five PKE transplants annually, leading to sparse arrivals and unstable parameter estimation if analyzed individually. Extending the time horizon beyond this period is not currently feasible, as no earlier PKE data exist and no additional cases were available at the time of data acquisition. Thus, these years were aggregated and analyzed as a single period to ensure robustness of the queueing model estimates. This period is sufficient to observe arrival processes, service completions, waiting‐time accumulation, utilization behavior, and episodes of queue stability and congestion, which are the central quantities of interest in queueing‐theoretic analysis.

**FIGURE 2:**
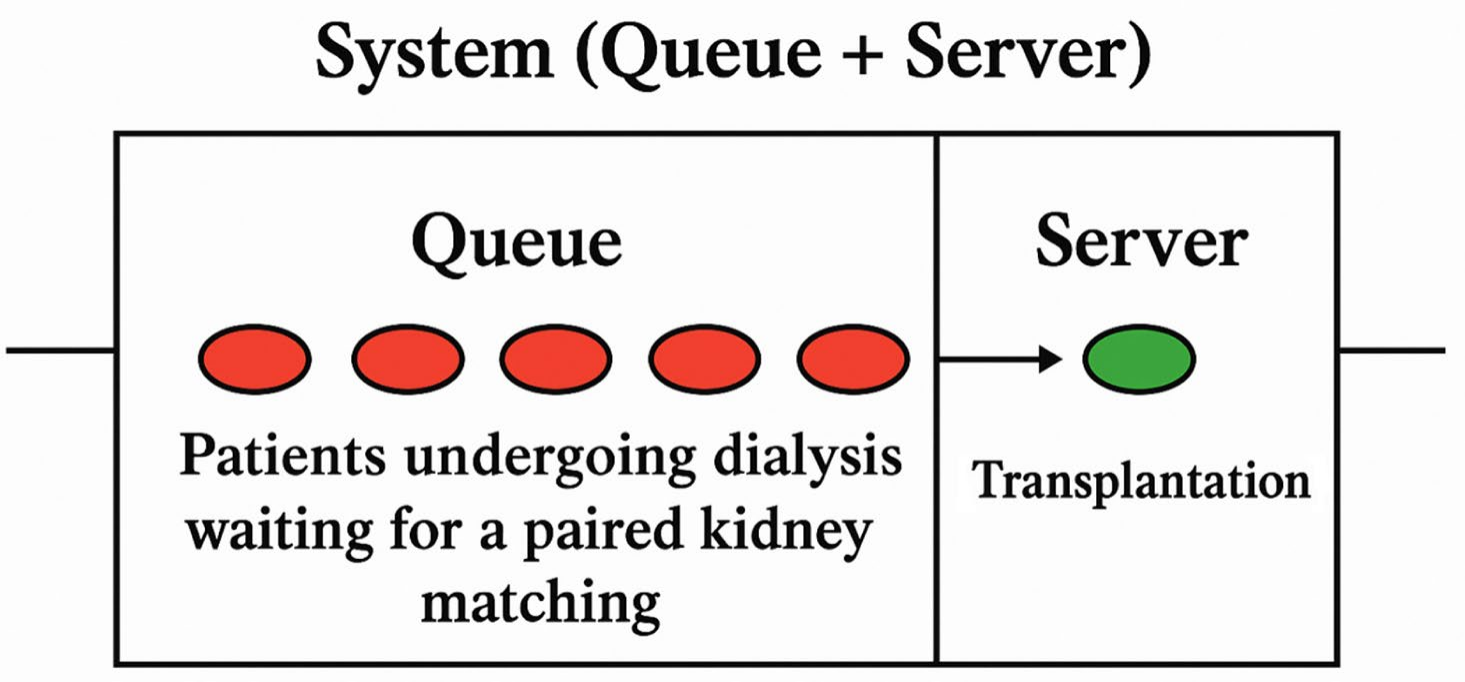
Queueing process of the transplant center.

Waiting time is measured from the diagnosis of ESRD, at which point kidney transplantation becomes the only definitive treatment option and dialysis is initiated, until successful transplantation. During this period, patients undergo donor compatibility testing with other potential donors in the pool while remaining on dialysis. Transplantation marks the service completion event in the queueing architecture, as illustrated in Figure 2. The sample size of the dataset consists of 34 (n_*d,r*_) incompatible donor‐recipient pairs which is further sorted into the aforementioned years based on the arrivals of the patients (and their incompatible donors) at the transplant facility to study the dynamics of the queue (2072, n_*d,r*_ = 3, 2073, n_*d,r*_ = 5, 2074, n_*d,r*_ = 11, 2075, n_*d,r*_ = 8, 2076–2077, n_*d,r*_ = 3, 2078–2080 n_*d,r*_ = 4). Each case corresponds to a single patient–donor registration at the time of entry into the PKE pool. The dataset includes only patients who ultimately received a successful transplant and information on patients who remained on the waiting list or were otherwise censored was not available from the transplant center, which represents a limitation of this study.

The dataset comprises all PKE transplants performed at the transplant center during the study period where only PKE cases are included. This captures the complete evolution of the national PKE queue from its inception. Conventional kidney transplants conducted within families or other closed donation arrangements are excluded, as the scope of this research is restricted to the dynamics of paired exchange queues. Nepal currently has only a handful of hospitals capable of kidney transplantation. Among these, the transplant center, which is the source of this study, is the only hospital in the country fully dedicated to organ transplantation and is the only facility in the country licensed to operate a formal PKE program. There are no spillover or latent effects from other hospitals that influence the queue dynamics of PKE analyzed in this study.

Given the small number of arrivals within each individual year, formal goodness‐of‐fit tests on individual years were not performed, as such tests are not robust with limited observations. Instead, this was assessed on the pooled inter‐arrival times across the entire observation period (34 transplants in total) as a diagnostic evaluation rather than a formal hypothesis test. Inter‐arrival times were assessed using a standard Kolmogorov–Smirnov (KS) test as a diagnostic check, since the first inter‐arrival is undefined and the data are sparse with extreme gaps, thus excluding the use of a Lilliefors correction. The KS statistic indicated some deviations from a perfect exponential distribution (n = 33, mean = 3.81 months, KS statistic = 0.306, p = 0.003), but the majority of inter‐arrival times follow the expected pattern. Despite the observed deviations in the inter‐arrival times, the patient arrivals can be reasonably approximated as an exponential distribution where the number of arriving incompatible donor‐recipient pairs per unit of time follows Poisson distribution. This is supported by the key characteristics of a Poisson process [27]. Arrivals of incompatible donor–recipient pairs are regarded as random draws from a large underlying population of ESRD patients evaluated at the transplant center that participate in the PKE. Independence is satisfied because the arrival of one incompatible donor‐recipient pair does not influence the likelihood of another such pair arriving and each patient's need for a transplant is unrelated to other patients and their incompatible donors. The assumption of a constant average rate is reasonable over the pooled observation period, as the average number of arrivals per unit time (months) remains relatively stable despite some variability between years. Even though some years have more arrivals and some less, the average number of arrivals of such pairs doesn't fluctuate wildly. Proportionality holds because the probability of an arrival occurring is directly proportional to the length of the interval considered where longer intervals naturally accommodate more potential arrivals. Finally, the criterion of negligible multiple events is met because in any sufficiently small‐time interval, the probability of two or more such pairs arriving simultaneously is essentially zero.

Service times were tested for exponentially using a Lilliefors‐corrected KS test to account for the estimated rate parameter (μ), confirming approximate exponential behavior (n = 34, mean = 17.85 months, KS statistic = 0.101, p = 0.848), consistent with the assumption of exponentially distributed service times. Based on the above diagnostic analysis, patient arrivals to the transplant center are modeled as a Poisson process, and service times are modeled as exponentially distributed. These analyses support the use of the M/M/1 model as a reasonable analytical approximation for evaluating operational performance for the observed data while acknowledging potential deviations due to sparse and variable arrivals.

For analytical purposes, patient arrivals are approximated by a Poisson process and service times by an exponential distribution. The Poisson arrival process is commonly used in healthcare systems to represent random, Markovian, that is, memoryless and independent arrival patterns of patients over time. In this study, the arrival of patients needing transplants is not related to other patient and not dependent on past arrivals or future incoming patients along with their non‐compatible donors, which follows a Poisson process. Similarly, since the transplant center aims to provide service to patients as soon as they arrive, the service times are modeled using an exponential distribution. The exponential distribution for service times reflects the unpredictability in the duration of finding another suitable match from other incompatible pairs participating in the program and transplant procedures, since donor organs becoming available is typically unpredictable. Nonetheless, the exponential service time assumption preserves the Markovian property of memorylessness that is, even if an incompatible pair is already waiting in the queue, a newly arrived incompatible pair be immediately matched and served if a compatible exchange with other such incompatible pairs is found regardless of the waiting time of the earlier pair. Likewise, the dataset of the PKE service is based upon a single transplant center where PKE for 2 cycles is conducted simultaneously at the same time by the same surgical team. This system, where patients are aided through one centralized system of all types of medical resources for surgical and post‐operative services, supports the use of a single‐server model. These assumptions provide a simplified yet powerful structure for analyzing congestion, wait times, and system performance in kidney exchange programs. The λ is the average arrival rate of patients in the transplant hospital and μ the corresponding mean service rate based upon the service time distribution provided by the hospital, respectively. The arrival rate is calculated as the reciprocal of the mean inter‐arrival time, which reflects the average time gap between successive patient arrivals. Conversely, the service rate is the reciprocal of the average service time, which, in this study, corresponds to the waiting time until transplantation, that is, the dialysis duration based on observed patient's records. The estimated arrival and service rates indicate λ > μ, implying that demand for transplantation exceeds the service capacity of the system. This condition corresponds to an overloaded (unstable) queue in which the waiting list is expected to grow over time rather than converge to a steady state. Standardized assumptions for the arrival and service processes, an approach practically observed in hospital queueing systems and well‐supported in the literature. Similar applications of M/M/1 queueing models have been reported in practical data‐based healthcare settings. Cho et al. [28] report the use of M/M/1 queueing models in data‐driven healthcare settings of electronic medical record (EMR) systems, where Poisson arrivals and exponential service times are applied to analyze patient waiting times and system performance, such as in outpatient departments of multiple hospitals. Given this overload condition, the classical steady‐state M/M/1 performance measures do not strictly exist in a theoretical sense. However, for interpretative purposes, to analyze the transplant system, these expressions are evaluated to provide an indicative scale of congestion and to illustrate the magnitude of imbalance between arrivals and service capacity under current system parameters. These values should therefore be interpreted as notional benchmarks rather than exact long‐run expectations. Thus, the mathematical model aligns with a real‐world scenario that fits the characteristics of an M/M/1 queue, for which the standard performance measures are:

$$\text{Average number of patients in the system}:L_{s}=\frac{\lambda }{\mu -\lambda }$$

$$\text{Average number of patients in the queue}:L_{q}=\frac{\lambda^{2}}{\mu \left(\mu -\lambda \right)}$$

$$\text{Average time that}\;a\;\text{patient spends in the system}:W_{s}=\frac{1}{\mu -\lambda }$$

$$\;\text{Average waiting time for}\;a\;\text{patient in the queue}:W_{q}=\frac{\lambda }{\mu \left(\mu -\lambda \right)}$$

## 3 Numerical Results and Interpretations

Based on the available data, performance measures were calculated using the classical M/M/1 queueing model and are summarized in Table 1. The table shows year‐wise values of arrival rate (λ), service rate (μ), average patients in the system (L_*s*_), in the queue (L_*q*_), average waiting time in the system (W_*s*_), and in the queue (W_*q*_). These measures form the basis for continuous learning in an LHS, providing evidence to guide operational improvements and policy decisions.

**TABLE 1:** Performance measures over years.

Year (B.S.)	λ	μ	L_*s*_	L_*q*_	W_*s*_	W_*q*_
Up to 2072	0.054	0.026	−1.942	−0.400	−35.842	−73.865
2073	0.486	0.227	−1.878	−4.017	−3.864	−8.265
2074	0.968	0.052	−1.057	−19.566	−1.092	−20.212
2075	0.357	0.050	−1.164	−8.263	−3.259	−23.140
2076–2077	0.172	0.049	−1.399	−4.906	−8.109	−28.442
2078–2080	0.124	0.097	−4.624	−5.900	−37.175	−47.431

Table 1 shows the year‐wise empirical values of average number of patients (L_*s*_) in the hospital, the average number of patients (L_*q*_) on dialysis, the average waiting time (W_*s*_) in the transplant center and the average waiting time (W_*q*_) on dialysis along with respective arrival and service rates.

**FIGURE 3:**
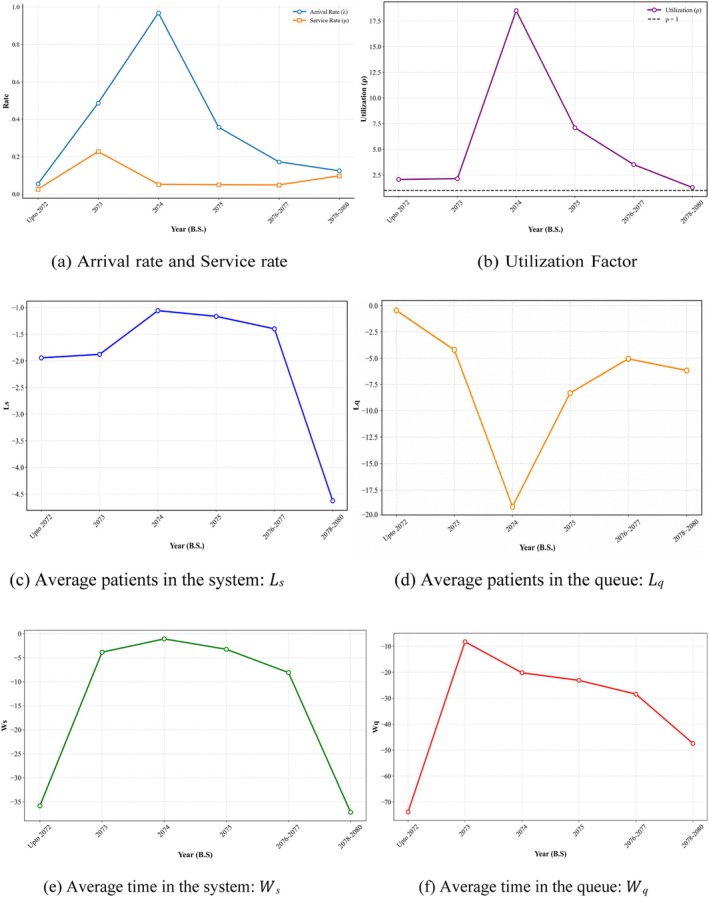
Performance measures of the PKE system. (a) Arrival rate and service rate. (b) Utilization factor. (c) Average patients in the system: *Ls*. (d) Average patients in the queue: *Lq*. (e) Average time in the system: *Ws*. (f) Average time in the queue: *Wq*.

The results obtained in Table 1 are graphically plotted. Here, Figure 3a,b indicate that arrivals of patients in the year 2074 B.S. are greater in comparison to any other year, which is greater than the service rate, λ > μ which is also seen in other years. These values do not represent true steady‐state expectations but provide an approximate scale of congestion under current system parameters. It is noted that in PKE, until a biological match is available for both incompatible donor‐recipient pairs, the patients are in the queue and till then the patients undergo dialysis as mentioned in Figure 2. Due to this condition, unstable queueing phenomena have been observed in all performance dynamics Figure 3c–f since incoming arrival rate is higher than service rate provided. This illustrates how data‐driven monitoring of patient flow, through queueing‐theoretic analysis, provides quantitative evidence of system instability and suggests the presence of operational constraints. The system is observed to operate in a predominantly congested regime across the majority of plausible parameter realizations. Rather than indicating deterministic instability, this reflects a high likelihood of congestion under realistic arrival and service conditions. While the magnitude of congestion varies, the qualitative behavior of the system remains consistent under parameter uncertainty.

The classical steady‐state M/M/1 formulations used above rely on the assumption that the system operates under equilibrium conditions (ρ <1). However, in the present study, the system is persistently overloaded (ρ >1), which violates these assumptions. As a result, some derived performance measures (e.g., queue length and waiting time) yield non‐physical values and are not directly interpretable in a real‐world context. To address this limitation, the analytical results are complemented with an empirical time‐average evaluation based directly on observed patient‐level data, which remains valid under non‐steady‐state and overloaded conditions. This study adopts an empirical time‐average technique based directly on observed patient‐level data. This approach avoids non‐physical artifacts (such as negative queue lengths and waiting times) arising from invalid steady‐state assumptions and instead relies on trajectory‐based measurements of system behavior. The empirical measures are:

Empirical average time in system:

$$W=\frac{1}{N}\sum_{i=1}^{N}w_{i}$$

where w_*i*_ denotes the waiting time of the *i*‐th patient, and N is the number of patients in the system during the observation period.

Empirical average system length:

$$L=\frac{1}{T}\int_{0}^{T}N\left(t\right)\;\mathrm{dt}$$

where $N\left(t\right)$ is the number of patients in the curve at time t, T is the total observation period (in months).

In practice, this is computed using the area under the system trajectory constructed from patient waiting‐time durations. Unlike steady‐state queueing formulas, these empirical measures do not require equilibrium assumptions and therefore provide physically meaningful estimates even when the system is persistently overloaded (ρ >1).

The empirical results reveal significant temporal variation in both waiting times and system occupancy, indicating a non‐steady‐state queueing process as shown in Table 2. Peak congestion is observed in 2074 (L = 17.50), while earlier and mid periods (2072, 2075) also show substantial backlog accumulation. In contrast, periods such as 2073 and 2078–2080 exhibit reduced congestion, reflecting partial system recovery. Notably, 2076–2077 demonstrates a regime of low occupancy but relatively high waiting time, suggesting that delays are influenced not only by queue length but also by service‐side constraints.

**TABLE 2:** Empirical time averages of the system.

Year	W (months)	*L* (average patients)
Up to 2072	38.00	9.50
2073	4.40	1.83
2074	18.18	17.50
2075	19.88	13.25
2076–77	20.33	2.54
2078–80	10.25	1.49

**FIGURE 4:**
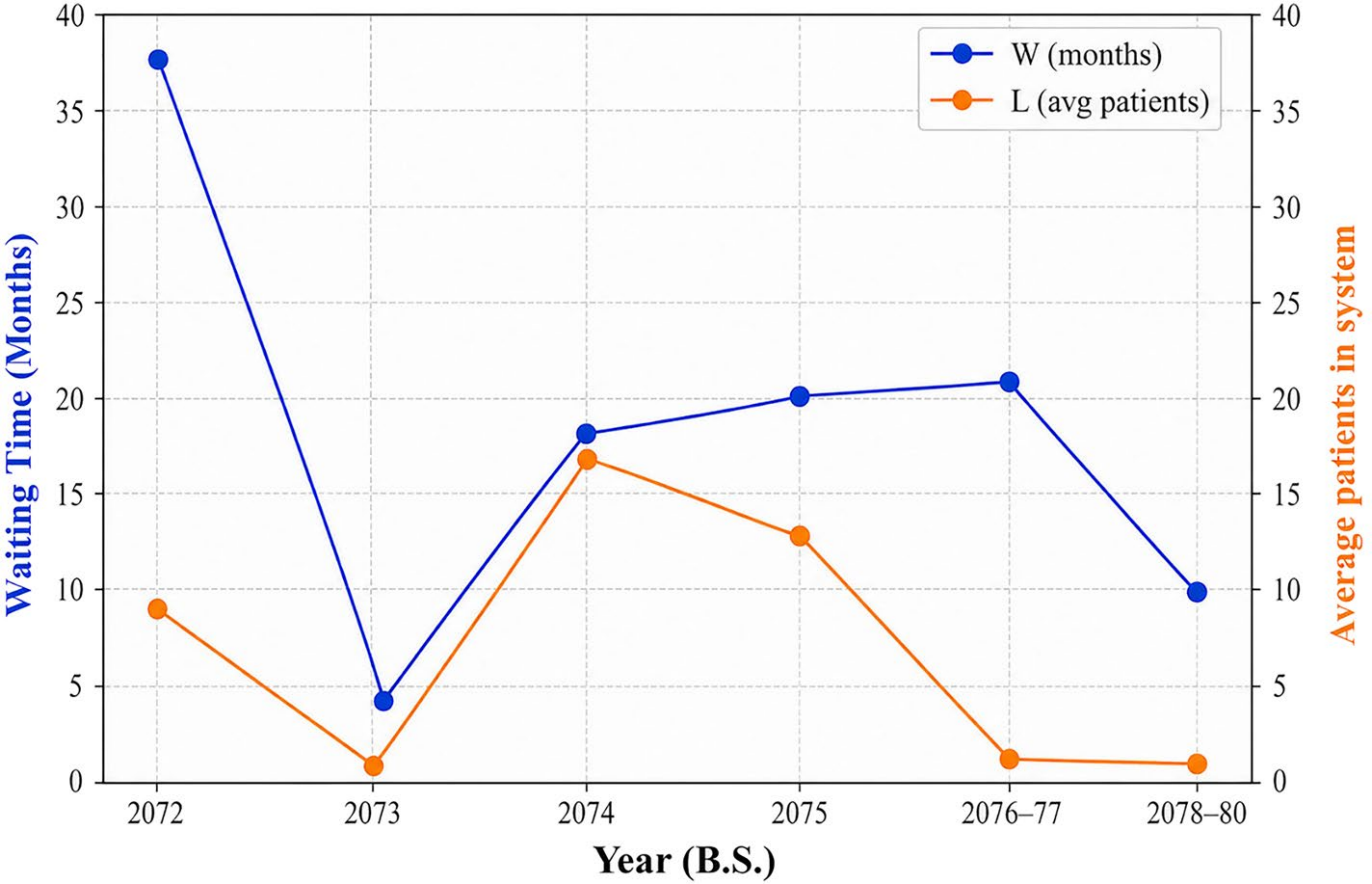
Empirical time‐average of the system.

Figure 4 illustrates the empirical time‐average performance of the system across different periods in terms of average waiting time (W) and average system occupancy (L). The results show clear temporal variability, indicating that system behavior is highly dynamic rather than steady‐state. The year 2074 exhibits the highest system occupancy (L = 17.50), reflecting a period of severe congestion where a large number of patients were simultaneously present in the system. Similarly, 2075 also shows elevated congestion levels (L = 13.25) with correspondingly high waiting times. In contrast, 2072 is characterized by the highest waiting time (W = 38.00 months) despite a comparatively moderate system size (L = 9.50), suggesting prolonged delays likely driven by slow matching or service inefficiencies rather than sheer queue buildup. Periods such as 2073 and 2078–2080 demonstrate relatively low system occupancy (L = 1.83 and 1.49, respectively) along with shorter waiting times, indicating phases of reduced demand or improved system flow. However, the 2076–2077 period presents a distinct pattern, where system occupancy remains low (L = 2.54) but waiting time is relatively high (W = 20.33 months). This suggests that congestion in this system is not solely determined by queue length, but is also significantly influenced by service‐side constraints, particularly delays in donor–recipient matching.

It highlights that both waiting time and system occupancy vary independently across periods, reinforcing that the system operates under non‐steady‐state conditions and that congestion is driven by a combination of demand intensity and compatibility‐driven service delays rather than queue length alone. While the primary performance measures have been interpreted with empirical time‐averages to ensure physical accuracy, the M/M/1 framework is retained for the sensitivity, correlation, and confidence interval analyses to serve as a standardized theoretical baseline for evaluating system responsiveness. This approach allows for the calculation of specific sensitivity coefficients and derivatives, which are essential for identifying which parameters arrival rate and service rate as the system architecture is most theoretically vulnerable to during periods of congestion. These secondary metrics provide a distinguishing view of the system's mathematical limits and asymptotic trends by illustrating the divergence between theoretical steady‐state expectations and the observed reality of a persistently overloaded regime. By maintaining this analytical strategy, the study remains grounded in established queueing literature while providing a comprehensive behavior of how theoretical vulnerabilities manifest in a practical, non‐equilibrium environment.

Table 3 reports year‐wise analytical confidence intervals for the arrival rate and service rate, and the corresponding implied bounds on system utilization (ρ). Instead of treating arrival and service rates as fixed numbers, 95% confidence intervals are calculated that capture plausible variation due to randomness and small sample sizes. Using exact methods ensures these intervals are accurate for the data, preventing overreliance on single‐point estimates and giving a more realistic picture of system behavior. Arrival rates were analyzed using exact Poisson confidence intervals, while service rates were analyzed using exact exponential confidence interval. The relatively wide confidence intervals observed for derived quantities such as utilization (ρ) are primarily attributable to the limited sample size, which introduces higher estimation uncertainty in the rate parameters. Using these bounds, system performance measures including ρ, L_*q*_, and W_*q*_ were recalculated to assess the sensitivity of congestion outcomes to uncertainty in λ and μ. The resulting analyses consistently suggested congestion across the plausible range of parameter estimates, indicating that the model‐based interpretation of operational congestion remained qualitatively stable despite uncertainty in the estimated rates. Collectively, the confidence interval analysis indicates that the model‐based interpretation of persistent operational congestion remains robust to parameter uncertainty, supporting the broader interpretation that biological compatibility constraints are consistent with being a major contributor to the observed operational congestion.

**TABLE 3:** Analytical confidence intervals (CI) of the system.

Year (B.S.)	CI (arrivals, λ)	CI (service, μ)	CI (utilization, ρ)	Congested?
Up to 2072	4.693 [0.618, 8.767]	0.023 [0.003, 0.042]	1211.47 [14.55, 2408.40]	Yes
2073	6.645 [1.623, 11.668]	0.081 [0.022, 0.139]	268.79 [11.60, 525.98]	Yes
2074	12.586 [5.492, 19.682]	0.006 [0.002, 0.009]	3748.69 [576.54, 6920.84]	Yes
2075	9.608 [3.453, 15.763]	0.009 [0.003, 0.014]	2378.50 [237.16, 4519.83]	Yes
2076–2077	4.693 [0.618, 8.767]	0.043 [0.006, 0.079]	648.92 [7.80, 1290.04]	Yes
2078–2080	5.6658 [1.089, 10.241]	0.050 [0.011, 0.089]	466.56 [12.18, 920.93]	Yes

It is important to note that the relatively wide confidence intervals observed for the derived performance measures reflect the uncertainty associated with the estimated queueing parameters and their propagation through the performance measures. Such conditions naturally increase the variance of estimated arrival and service rates, which propagates into wider uncertainty bounds for derived queueing measures. Accordingly, these intervals reflect statistical uncertainty arising from sparse data rather than instability in the underlying queueing model. Therefore, the purpose of the confidence intervals is not only to provide precise point estimates of system performance, but also to describe a plausible range of system behavior under data‐limited conditions.

**FIGURE 5:**
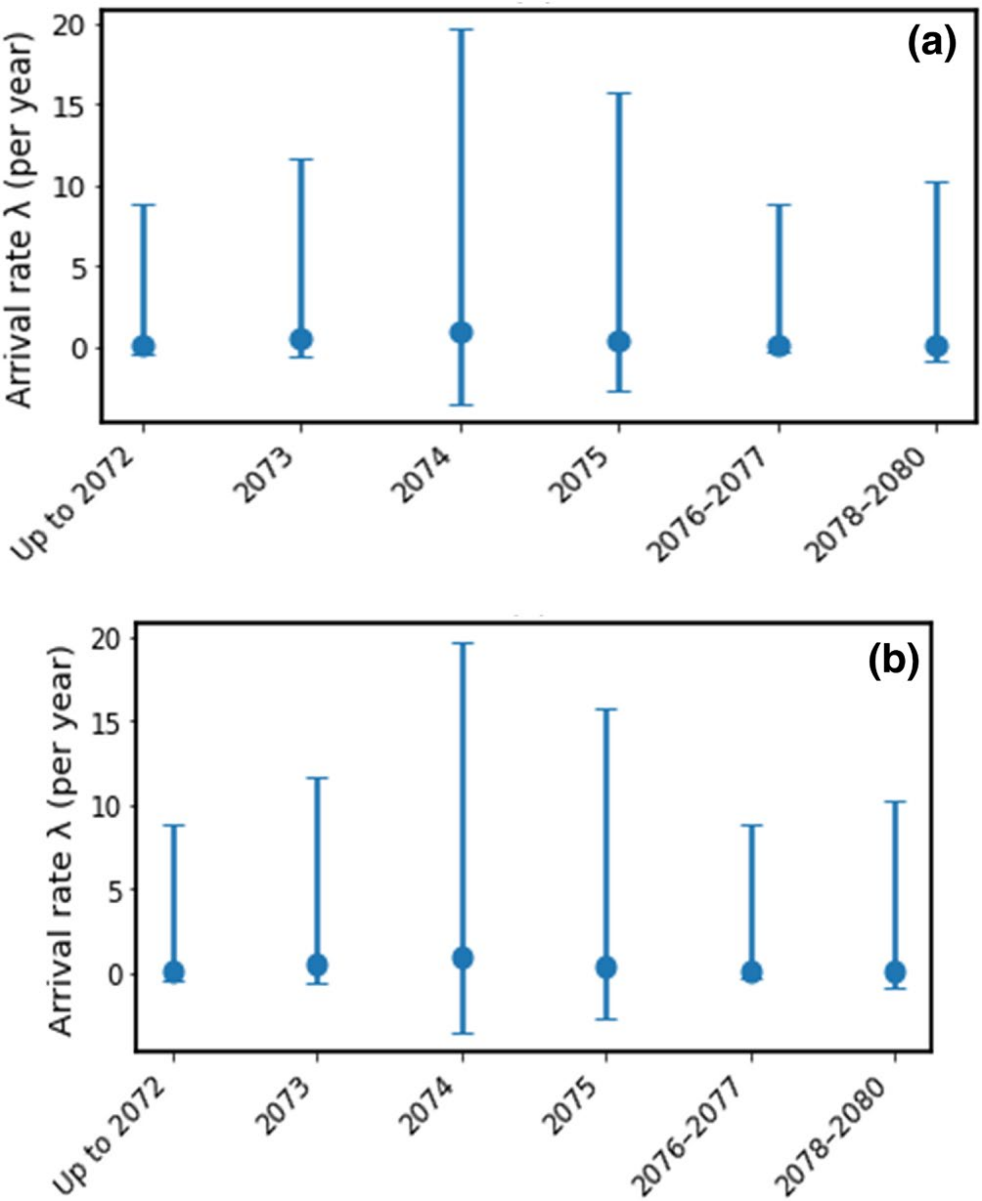
Analytical confidence interval analysis of the system. (a) Arrival rate with 95% CI. (b) Service rate with 95% CI.

Figure 5a reports the arrival rates with 95% confidence intervals to reflect uncertainty due to sampling variability. Queueing outcomes are evaluated across this entire range to assess the sensitivity of system performance to plausible variation in arrival intensity. Notably, even under conservative assumptions represented by the lower confidence bounds, arrival rates remain high enough to sustain congestion. These findings suggest that the model‐based interpretation of operational congestion is qualitatively stable across the plausible range of arrival rates and provide evidence consistent with operational congestion arising from service capacity limitations and biological matching constraints rather than being solely attributable to random fluctuations in demand. Figure 5b presents service‐rate estimates together with 95% confidence intervals, reflecting uncertainty in system processing capacity due to finite‐sample estimation. Rather than fixing μ at its point estimate, queueing behavior is examined across this range to assess sensitivity to plausible improvements in service performance. Even under the most favorable capacity assumptions implied by the upper confidence bounds, utilization remains elevated relative to observed arrival rates. This suggests that congestion persists despite optimistic service conditions, highlighting a fundamental capacity shortfall rather than inefficiencies in service provision. While confidence intervals are visualized for the arrival and service processes, utilization, ρ is summarized numerically rather than graphically. Since ρ is a derived quantity defined as the ratio of arrival and service rates, its uncertainty is fully characterized by the confidence intervals of λ and μ. Given the extreme skewness of these intervals, tabulation provides a clearer summary while still demonstrating that congestion persists across all plausible parameter values. Even across plausible variations in arrival and service rates, utilization remains high, showing that congestion arises from the structural constraint that patients can only be matched with compatible donors, rather than from statistical uncertainty.

Although the preceding analyses provide a comprehensive evaluation of queueing performance and its associated uncertainty through analytical confidence intervals, these estimates remain sensitive to the limited sample size. To further assess the robustness of the estimated queueing parameters, uncertainty was additionally evaluated using the Hutson‐B bootstrap, a smooth resampling method designed to improve interval estimation in small‐sample settings. Among the bootstrap procedures evaluated by Simkus et al. [29], the Hutson‐B and Banks‐B methods demonstrated robust coverage and parameter estimation performance, whereas the conventional Efron bootstrap generally exhibited inferior coverage accuracy. Since the primary objective of the present study was reliable evaluation of queueing parameters rather than prediction of future observations, the Hutson‐B bootstrap was selected as an appropriate complementary inferential approach.

The Hutson‐B bootstrap was applied to the observed arrival and service data using 10 000 bootstrap replications to obtain bootstrap estimates and 95% confidence intervals for the arrival rate, service rate, utilization, and the derived queueing performance measures. Unlike the analytical confidence intervals, which rely on theoretical sampling distributions, the Hutson‐B bootstrap estimates parameter uncertainty through smooth resampling of the observed data. Consequently, it provides an independent robustness check for the analytical confidence interval results presented in the preceding sections. By generating smooth bootstrap samples using a composite quantile estimator, the method produces continuous bootstrap distributions that enable estimation of the uncertainty associated with the queueing parameters. This resampling‐based approach complements the analytical method without relying on distribution‐based variance formulas.

Table 4 presents the Hutson‐B bootstrap estimates together with their corresponding 95% confidence intervals for the arrival rate, service rate, and system utilization. Across all study periods, the bootstrap confidence intervals revealed substantial variability in the estimated arrival rates, service rates, and utilization, reflecting both the number of observed transplant cases and the variability of the underlying arrival and service processes. Wider confidence intervals were generally observed during study periods with fewer transplant cases, whereas comparatively narrower intervals were obtained for periods with more observations or lower variability in the underlying data. Despite this variability, the bootstrap analysis consistently yielded utilization estimates exceeding one across all study periods, indicating that the qualitative interpretation of persistent operational congestion remained unchanged. The close agreement between the analytical and bootstrap confidence interval analyses further demonstrates that the observed uncertainty is consistently reflected across two complementary inferential approaches, thereby strengthening the robustness of the study's overall interpretation while acknowledging the limitations imposed by the available data.

**TABLE 4:** Hutson‐B bootstrap estimates of queueing parameters with 95% confidence intervals.

Year (B.S.)	CI (arrivals, λ)	CI (service, μ)	CI (utilization, ρ)	Congested?
Up to 2072	0.166 [0.028–0.862]	0.035 [0.014–0.130]	6.29 [0.37–43.10]	Yes
2073	0.544 [0.246–1.206]	0.253 [0.137–0.540]	2.45 [0.67–6.39]	Yes
2074	1.050 [0.449–2.529]	0.055 [0.031–0.104]	20.98 [6.53–55.39]	Yes
2075	0.396 [0.181–0.957]	0.053 [0.032–0.097]	8.02 [2.77–21.03]	Yes
2076–2077	0.173 [0.157–0.191]	0.051 [0.042–0.071]	3.45 [2.43–4.47]	Yes
2078–2080	0.224 [0.049–0.852]	0.051 [0.051–0.408]	2.38 [0.24–12.05]	Yes

**FIGURE 6:**
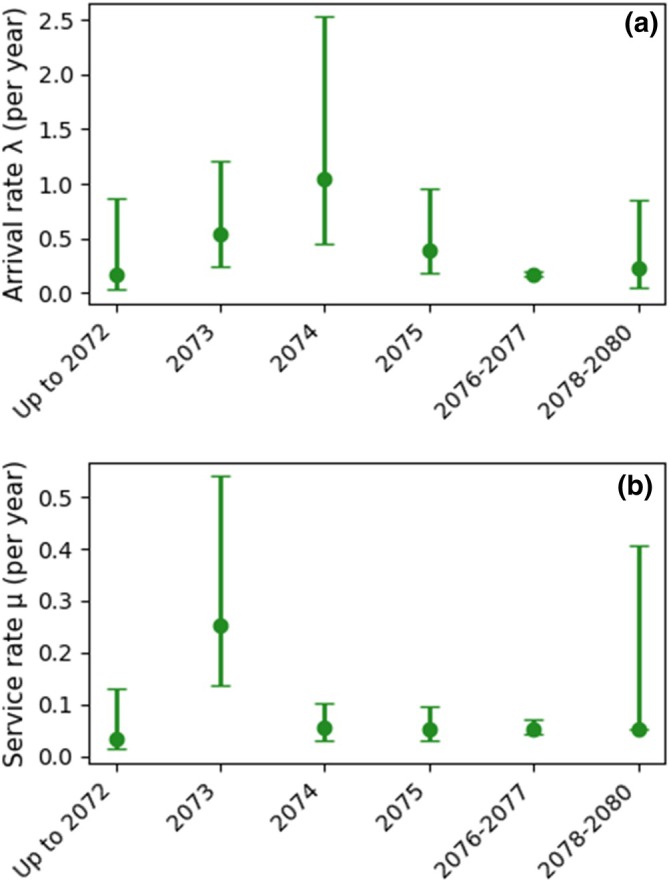
Confidence interval analysis with Huston‐B bootstrap estimates.(a) Bootstrap arrival rate with 95% CI. (b) Bootstrap service rate with 95%.

Figure 6a illustrates the Hutson‐B bootstrap estimates of λ together with their 95% confidence intervals across the study periods. The estimated arrival rates varied over time, with noticeable differences in the precision of the estimates. Relatively wide confidence intervals were observed for the up to 2072, 2073, 2074, 2075, and 2078–2080 periods, reflecting greater uncertainty associated with the limited number of observed transplant cases and variability in the arrival process. In contrast, the 2076–2077 period exhibited a comparatively narrow confidence interval, indicating a more precise estimate of the arrival rate during that period. Figure 6b presents the Hutson‐B bootstrap estimates of the μ together with their corresponding 95% confidence intervals. Similar temporal variation was observed in the estimated service rates. The confidence intervals were relatively wide for the up to 2072, 2073, and 2078–2080 periods, indicating greater uncertainty in the estimated service rates, whereas the 2074, 2075, and 2076–2077 periods exhibited comparatively narrower confidence intervals, suggesting more precise estimation during these study periods. These bootstrap confidence intervals demonstrate that the precision of the estimated arrival and service rates varied considerably across the study periods and was strongly influenced by the number of observed transplant cases and the variability of the underlying arrival and service processes.

Overall, the Hutson‐B bootstrap confidence intervals were broadly consistent with the results of analytical confidence intervals (Figure 5a,b), as both approaches produced relatively wide intervals for several study periods and suggest system congestion. This indicates that the observed uncertainty is not an artifact of relying solely on analytically derived confidence intervals but is consistently reflected across two complementary methods of uncertainty estimation. Furthermore, both approaches yielded the same interpretation of persistent operational congestion despite differences in the numerical values and widths of the estimated confidence intervals. Consequently, the Hutson‐B bootstrap strengthens the robustness of the analysis while reinforcing that the reported queueing measures are model‐based estimates subject to uncertainty rather than as precise population‐level quantities. As the available transplant data comprise a relatively small sample, both the analytical and bootstrap approaches produced wide confidence intervals for several parameters, particularly system utilization, reflecting the inherent uncertainty associated with estimating queueing parameters from limited observations. Accordingly, the bootstrap analysis is a complementary robustness assessment of parameter uncertainty rather than a means of overcoming the limitations imposed by the available data.

Confidence intervals provide a quantitative basis for risk‐aware planning. In an LHS, stakeholders can use these bounds to anticipate worst‐case congestion scenarios, allocate resources proactively, and evaluate policies under uncertainty, rather than relying on single point estimates that underestimates stress on the system. Despite the increased uncertainty reflected in wider confidence bounds, particularly for derived measures such as ρ due to the small sample size impression, the direction and presence of congestion remain consistent across all evaluated scenarios. Furthermore, a descriptive correlation analysis of the computed metrics is analyzed to assess the nature and degree of the linear relationship between average patients in the queue and other queueing terminologies.

**FIGURE 7:**
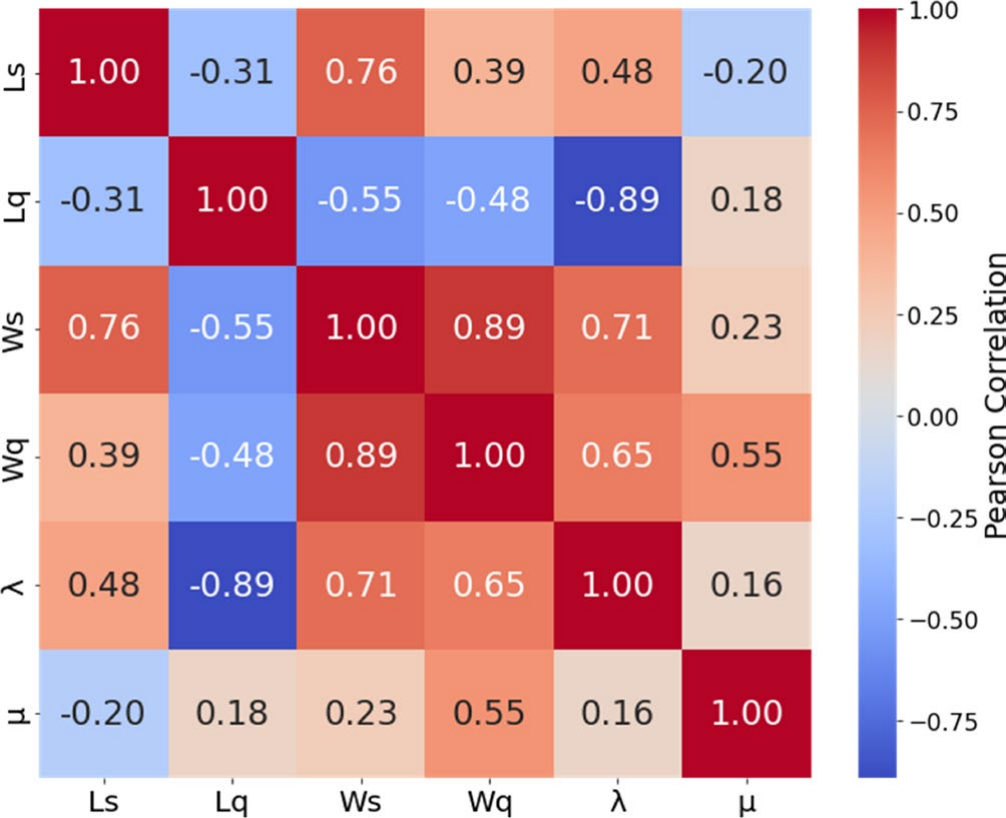
Correlation heatmap of performance measures of the system.

Figure 7 represents the correlation heatmap illustrating the relationships among the arrival rate, service rate, and average number of patients in the system, average number of patients in the queue, average waiting time in the system, and average waiting time in the queue. A negative correlation was observed between Lq and Ls (r= −0.31), representing an unusual relationship relative to the expectations of the classical M/M/1 queueing model. This pattern is consistent with the operational characteristics of the studied PKE system, in which patients remain on the waiting list until a biologically compatible donor–recipient match is identified, and increases in the number of patients awaiting transplantation do not necessarily correspond to proportional increases in completed transplants. Consequently, waiting times may remain prolonged despite variations in system occupancy.

Similarly, Lq exhibited negative correlations with both Ws (r= −0.55) and Wq (r= −0.48). These counterintuitive relationships differ from those typically expected in classical queueing systems and may reflect the influence of compatibility‐constrained donor–recipient matching, where waiting time is not determined solely by queue length but also by the availability of biologically compatible matches. Likewise, the negative correlation between the arrival rate and Lq (r = −0.89) contrasts with the conventional expectation that higher arrival rates are associated with longer queues. In the studied PKE system, however, increases in patient arrivals do not necessarily translate into proportional increases in completed transplants because transplantation remains conditional on identifying biologically compatible donor–recipient pairs. Accordingly, these observed correlations are consistent with the compatibility‐driven nature of the matching process under the M/M/1 system.

A weak positive correlation (r = 0.18) was observed between the service rate and Lq, which likewise differs from the relationship expected under the classical M/M/1 model, where higher service rates generally reduce queue length. In the context of paired kidney exchange, however, improvements in nominal service capacity alone may not substantially reduce congestion when biologically compatible donor–recipient matches remain limited. Consequently, patients may continue to experience prolonged waiting times despite available clinical and surgical capacity. Collectively, these correlation patterns suggest that biologically constrained healthcare systems such as PKE may exhibit operational behavior that differs from the assumptions of classical queueing models, highlighting the value of iterative learning and continuous operational evaluation within a Learning Health System framework.

The classical M/M/1 queueing model assume a stochastic service process with continuous and independent service completions. However, in the present system of dialysis‐based transplant waiting process, patient departure from the queue is not governed solely by mathematical service rate dynamics but by the availability of a biologically compatible donor kidney and subsequent transplantation (Figure 2). This introduces an inherent biological matching constraint, which makes service completion conditional rather than continuous, within the available pool, fundamentally altering the queueing process. Consequently, service completion is conditional and irregular, resulting in prolonged waiting times and backlog accumulation even under moderate arrival rates. As a result, even when arrival rates are relatively low, limited donor–recipient compatibility leads to prolonged waiting times and accumulation of backlog. Thus, when the system operates in the regime ρ > 1, the M/M/1 model indicates mathematical instability, manifested as unbounded queue growth which are retained not as physically bounded performance measures, but as indicators of congestion intensity and structural imbalance between demand and service capacity.

To assess the sensitivity of the model‐based interpretation of system congestion to plausible variation in the estimated arrival and service rates by ±10% and ±20%, reflecting plausible deviations from the observed averages. For each combination of the parameters, the corresponding system utilization (ρ) was calculated as shown in Table 5. Under the ±10% variation, all yearly subsystems of individual years remained congested, yielding ρ > 1. Under the wider ±20% variation, all years except the 2078–2080 period remained congested with the most optimistic scenario resulted in ρ ≈ 0.85 (ρ< 1), indicating that, under favorable realizations of the arrival and service rates within their uncertainty ranges, the system operates in a stable (non‐congested) state. This conditional stability observed only for the 2078–2080 period under sensitivity analysis reflect the COVID‐19 based lockdown that induced reduction in patient arrivals, which likely eased system congestion, allowing the transplant center administration additional time to review donor–recipient registries, complete matching procedures, and proceed with PKE transplantation. Overall, these results, considered alongside the exact 95% confidence intervals for λ and μ, support the model‐based interpretation that persistent congestion remains qualitatively consistent across plausible variations in the estimated parameters. Importantly, these findings suggest that even under modest alternative assumptions, the M/M/1 queueing model provides a useful analytical framework for characterizing the overloaded behavior of the studied system.

**TABLE 5:** Sensitivity analysis of the system.

Year (B.S.)	ρ	ρ_min_ (±10%)	ρ_max_ (±10%)	ρ_min_ (±20%)	ρ_max_ (±20%)	Congested ±10%?	Congested ±20%?	Minimum increase in μ for stability %
Up to 2072	2.061	1.686	2.519	1.374	3.091	Yes	Yes	106.1
2073	2.139	1.750	2.616	1.426	3.209	Yes	Yes	113.9
2074	18.508	15.143	22.621	12.339	27.763	Yes	Yes	1750.8
2075	7.099	5.809	8.677	4.733	10.649	Yes	Yes	609.9
2076–2077	3.513	2.874	4.294	2.342	5.270	Yes	Yes	251.3
2078–2080	1.276	1.043	1.559	0.851	1.914	Yes	No (possible)	27.6

**FIGURE 8:**
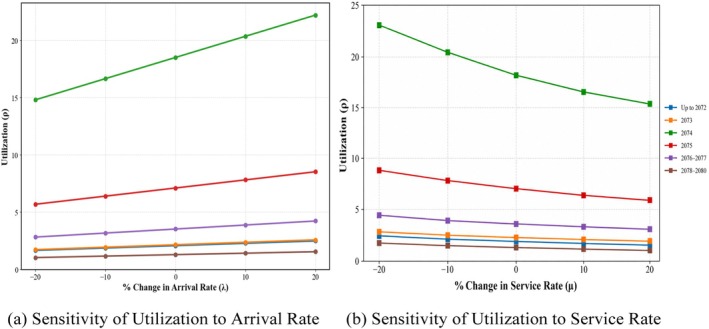
Sensitivity analysis of the system. (a) Sensitivity of utilization to arrival rate. (b) Sensitivity of utilization to service rate.

Figure 8a,b depict the sensitivity of ρ to parameter estimates in λ and μ, respectively. Consistent with the sensitivity results summarized in Table 3, it is consistent with the observation that, once the system is congested (ρ > 1), higher arrival rates are associated with prolonged effective service times within the modeling outline. While higher arrival rates mechanically increase utilization, the pronounced sensitivity to reductions in service capacity suggests that increased arrival pressure may contribute to delays in the matching process. Consequently, under the model, service completion may be delayed even when nominal service capacity remains unchanged. This indicates that, in an inherently complex matching environment, increases in arrivals intensify congestion and hinder timely matching, thereby effectively increasing service time and preventing the system from clearing demand. As arrival rates increase, the system experiences delay even with unchanged service capacity, as each donor–recipient match takes longer due to queue buildup. Elevated arrivals complicate congestion, hinder timely matching, and prevent the system from processing all patients efficiently. These figures illustrate that the qualitative pattern of congestion remains consistent across the evaluated parameter variations. Thus, as shown in Table 3 and confidence interval bounds followed by sensitivity analysis in Table 5, the model‐based interpretation of persistent congestion remains qualitatively consistent across the evaluated parameter variations.

Taken together, the sensitivity analysis provides evidence consistent with the compatibility‐driven matching process being a major operational constraint in the studied PKE system. Even when arrival rates or service rates are varied within ±10% or ±20%, the system remains congested in most years, indicating that delays are not simply due to temporary fluctuations in demand or service capacity. This is consistent with the structural limitation that each patient and donor can only be matched with compatible pairs, which inherently restricts throughput. By systematically evaluating how small changes in λ and μ affect utilization, queue length, and waiting time, the analysis suggests that compatibility‐driven matching, rather than service capacity alone, is a major contributor to prolonged waiting times and queue buildup, providing quantitative evidence consistent with its role as a major operational constraints in the studied PKE system. Figure 8 represents structural sensitivity of system utilization to variations in arrival and service rates, rather than statistical estimation uncertainty. Since these plots are derived from deterministic transformations of λ and μ, the uncertainty associated with parameter estimation is captured. As a result, additional confidence intervals or standard errors are not directly applicable to Figure 8.

To complement the sensitivity analysis, the minimum increase in service capacity (μ) required to achieve system stability (ρ ≤ 1) under baseline arrival rates are estimated. Since, ρ = λ\μ, stability requires ρ ≤ 1 ⇒ μ ≥ λ. Therefore, the required percentage increase in service capacity can be expressed as % increase in *μ*. The results indicate that substantial capacity expansion is required in almost all periods, particularly in earlier years where utilization is significantly above unity. For instance, as mentioned in Table 5, the 2074 B.S. period would require an increase of approximately 1751% in service capacity to stabilize the system, reflecting extreme congestion conditions. In contrast, the 2078–2080 period requires only a modest increase of approximately 27.6%, consistent with reduced demand conditions associated with the COVID‐19‐related disruption. These results are consistent with congestion being largely associated with structural constraints and suggesting that achieving stability through service expansion alone may be impractical in most study periods.

From an LHS perspective, the sensitivity analysis shows that the model‐based interpretation of congestion remains qualitatively consistent. This highlights the need for adaptive, data‐driven strategies that prioritize managing demand such as scheduling or optimizing donor‐recipient matching over marginal increases in service capacity. By systematically evaluating how small changes in λ and μ affect utilization, queue length, and waiting time, the analysis provides evidence consistent with compatibility‐driven matching being a major contributor of congestion leading to prolonged waiting times and queue buildup in the studied PKE system.

Additionally, linear regression is performed as an inferential statistical technique to model how arrival and service rates influence queue lengths and waiting times.

**FIGURE 9:**
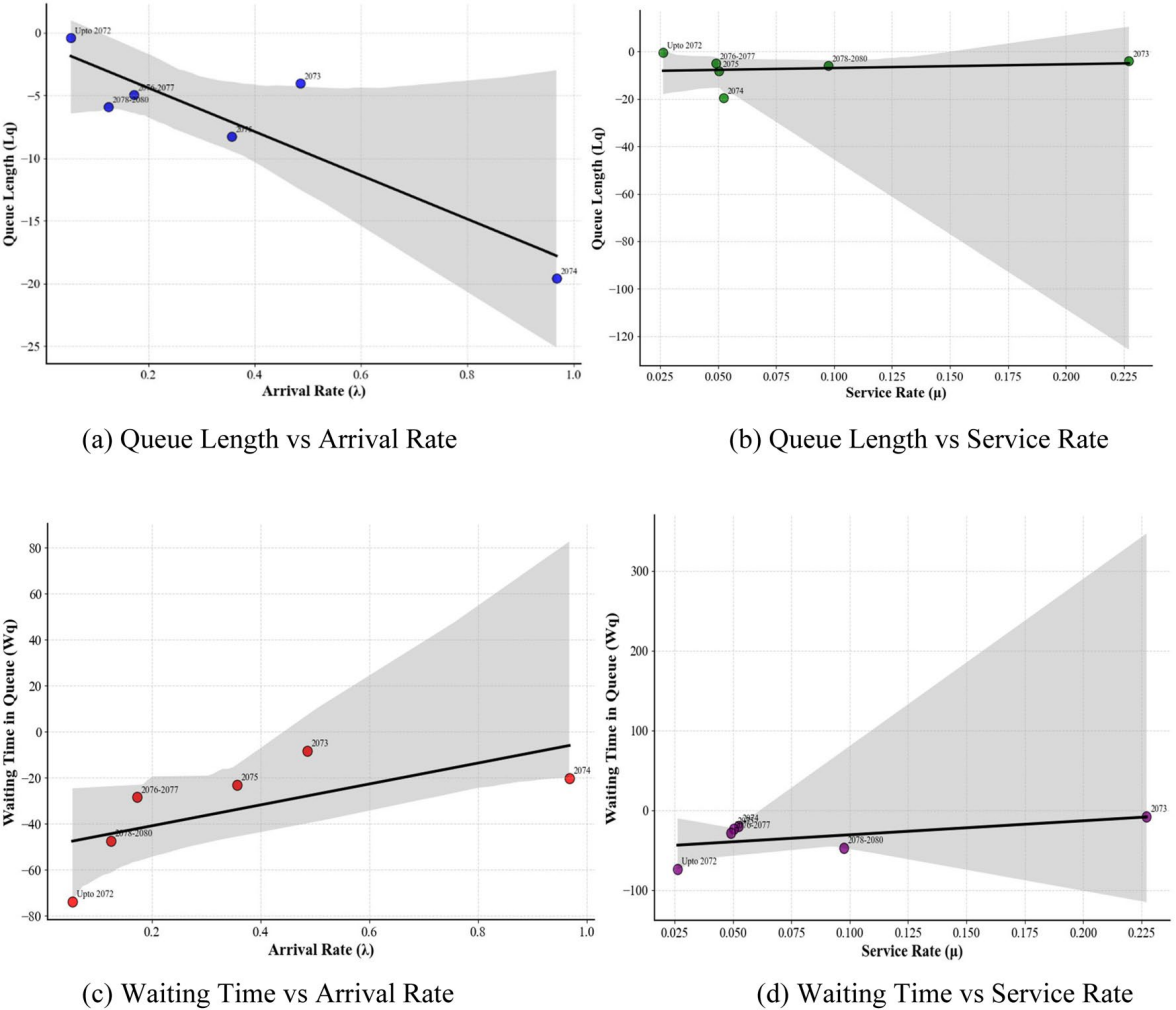
Linear regression analysis of the system. (a) Queue length versus arrival rate. (b) Queue length versus service rate. (c) Waiting time versus arrival rate. (d) Waiting time versus service rate.

A similar interpretation was obtained from the linear regression analysis. In Figure 9a there is an unusual relationship that differs from the behavior predicted under the classical steady‐state M/M/1 model. In periods of higher estimated arrival rates, the observed queue length increased substantially. Figure 9b suggests that increases in nominal service rate alone were not associated with proportional reductions in congestion. This trend leads to a continued influx of patients, contributing to a growing waitlist and increasing the system's instability.

Figure 9c represents that as the arrival rate is increasing, the waiting time for the patients is also increasing, which differs from the classic queueing theoretical scenario. Similarly, Figure 9d illustrates that a slight increase in the service rate is associated with an increase in queue waiting time. Rather than representing a contradiction to queueing theory, this behavior reflects the biological compatibility constraint of paired kidney exchange, where transplantation cannot proceed until a feasible donor–recipient match is identified as illustrated in Figure 2. Consequently, increasing service capacity alone may not reduce waiting time when matching remains the dominant constraint. The regression and correlation analyses together suggest that biologically constrained queueing systems such as PKE may exhibit behavior that differs from the assumptions of classical queueing models; biologically constrained queueing systems like PKE may exhibit persistent congestion arising from compatibility‐driven operational constraints, emphasizing the importance for healthcare administrators and policymakers to adopt forward‐looking interventions aimed at minimizing potential future disruptions.

Regression summaries are presented in Table 6 where the results indicate a strong positive association between L_*q*_ and λ and between W_*q*_ and λ, demonstrating that increases in arrival rate substantially amplify both queue length and waiting time. These findings are consistent with queueing theory, where congestion metrics are highly sensitive to traffic intensity. In contrast, the associations between congestion measures and service rate μ are weak and statistically insignificant L_*q*_ and W_*q*_ and μ, indicating that variations in service capacity within the observed range have limited impact on reducing congestion. This is consistent with the operational characteristics of the studied PKE system, the number of available matches and processing resources is relatively constrained, so even moderate improvements in service rate are insufficient to offset high arrival pressure from incompatible donor–recipient pairs. Overall, these regression analyses provide complementary statistical evidence supporting the queueing‐based interpretation and are consistent with arrival pressure and biological compatibility constraints being major contributors to congestion in the studied PKE system.

**TABLE 6:** Regression summary.

Parameter	Intercept	Intercept error	Slope	Slope error	*R* ^2^	p
L_*q*_ vs. λ	−0.171	0.323	3.065	0.681	0.835	0.010
L_*q*_ vs. μ	1.197	0.806	−3.155	7.488	0.042	0.695
W_*q*_ vs. λ	−0.171	0.367	3.529	0.775	0.838	0.010
W_*q*_ vs. μ	1.409	0.925	−3.699	8.595	0.044	0.689

Analysis of the PKE system using confidence intervals, sensitivity testing, and regression modeling consistently highlights persistent congestion as a qualitatively consistent feature of the model‐based analyses. Confidence intervals for the arrival and service rates quantify plausible variability arising from stochastic fluctuations and limited sample sizes. Even under conservative lower‐bound estimates, the arrival rates remain sufficiently high to sustain congestion, suggesting that the observed operational congestion is not solely attributable to statistical uncertainty in the estimated parameters. Sensitivity analysis further suggests that modest deviations in λ or μ, within ±10% or ±20%, do not materially alter system utilization (ρ> 1 for most years), illustrating that congestion persists across plausible operational conditions. Regression analyses complement these findings by indicating, revealing a strong positive association between queue length and waiting time with arrival rates and service rates while the associations with service rates are weak and statistically insignificant, reflecting the limited ability of nominal service capacity to offset high arrival pressure from incompatible donor–recipient pairs. Collectively, the queueing, confidence interval, sensitivity, regression, and correlation analyses provide evidence consistent with biological compatibility requirements for donor–recipient matching being a major operational constraint contributing to congestion in the studied PKE system with the M/M/1 model. Patients remain in the queue until suitable donor‐recipient matches are found, causing persistent congestion. Thus, the analyses suggest that compatibility‐constrained matching is an important contributor to prolonged waiting times within the studied system.

From an LHS perspective, these results collectively indicate that adaptive, data‐driven strategies should focus on managing arrivals and optimizing matching processes, rather than solely increasing service capacity. Continuous monitoring of λ, μ, and the resulting congestion metrics enables the queueing‐theoretic framework to support the evaluation of potential operational challenges, guide iterative service improvements, and inform targeted interventions, consistent with LHS principles of continuous feedback, learning, and data‐driven decision‐making.

These insights highlight the importance of continuous learning within an LHS, where unusual trends in operational metrics can guide step‐by‐step improvements in resource use and patient care strategies. By uncovering patterns that initially appear counterintuitive, the results provide quantitative evidence for evaluating constraints in the PKE system, with the resulting insights feeding back into planning and policy updates in accordance with the core principles of a Learning Health System. Recognizing this structural constraint allows healthcare managers and policymakers to prioritize interventions such as improving donor‐recipient matching processes, expanding registry coverage, and optimizing scheduling. Feeding these evidence‐based insights back into planning and policy updates creates a data‐driven feedback loop that strengthens equity, efficiency, and quality of care. Embedding such findings into the LHS cycle enables ongoing evaluation, adaptive policy changes, and informed operational decisions to reduce waiting times and improve transplant outcomes.

## 4 Conclusion and Discussions

This study applied an M/M/1 queue model from the real anonymized data from a transplant center, considering dialysis duration as the waiting period for the respective recipients and the transplant date as the time when the patients get service. The findings from the analysis indicate that the overall queue behaves randomly, and the system is significantly congested, evaluating operational stress in the transplant system. Such overload in the system, signifies a hefty increase in renal patients with end‐stage renal disease who are longing for a transplantation. This resulted in prolonged waiting times for recipients to receive kidneys along with their paired donors to gain a match and has significantly affected the lives of many such patients. By applying queueing theory to a real‐world healthcare environment, this study demonstrates the use of mathematical modeling to generate actionable insights, feeding evidence back into practice and policy to improve care delivery within LHS paradigm.

These findings also reflect observable signs of lack of coordination in the stressed system, particularly due to structural constraints in donor‐recipient matching, where increases in arrival rates do not directly translate into increased transplants, highlighting system‐based inefficiencies inherent in the biologically constrained process. The robustness of these findings is further reinforced by confidence interval analysis, which shows that even under the lower bounds of arrival and service rates, utilization remains high across all years. Sensitivity analyses varying arrival and service rates by ±10% and ±20% confirm that congestion persists under plausible fluctuations in system parameters. Regression analysis further supports this, demonstrating that queue length and waiting times are strongly correlated with arrival rates, whereas variations in service rate alone have limited impact. Taken together, these analyses provide evidence consistent with biological compatibility constraints governing donor–recipient matching being a major operational contributor to the observed congestion in the adopted M/M/1 system, rather than stochastic fluctuations or temporary variations in service capacity.

Even when surgical and clinical capacity is fully available, transplantation cannot proceed without identifying a biologically compatible donor. In paired kidney exchange, this requirement represents a fundamental operational constraint that may leave patients on prolonged dialysis while awaiting a feasible match. Furthermore, delays in transplantation may arise not only from donor scarcity but also from the combinatorial complexity of identifying biologically compatible donor–recipient pairs within exchange cycles or chains, making timely matching a significant operational challenge. The findings of the present study suggest that biological compatibility constraints contribute substantially to observed delays in the matching process. This interpretation is based on the collective evidence obtained from the complementary analytical, simulation, and queueing analyses presented in this study, rather than on any single performance measure. Specifically, the queueing analysis consistently estimated arrival rates exceeding service rates (λ >μ), resulting in utilization greater than one and prolonged waiting times. Both the analytical and Hutson‐B bootstrap confidence interval analyses demonstrated that these findings remained qualitatively consistent despite parameter uncertainty. Sensitivity analyses further showed that plausible variations in arrival and service rates did not substantially alleviate congestion, while regression and correlation analyses indicated that queue length and waiting times were predominantly associated with arrival pressure, with changes in service rate alone having comparatively limited influence. Collectively, these analyses provide evidence consistent with biological compatibility constraints in donor–recipient matching being a major operational contributor to the observed congestion. With this, the estimated queueing measures consistently indicate operational conditions compatible with an overloaded system. Accordingly, the combined analytical findings suggest that the observed operational congestion is consistent with an important contribution from structural biological compatibility constraints under the M/M/1 system.

While this research applies an M/M/1 queueing model to evaluate operational performance in the PKE program using real‐world transplant data, the interpretation of the estimated queueing measures is necessarily constrained by the scope of the available dataset. Specifically, only records from 34 incompatible donor–recipient pairs who successfully completed PKE cycles since the initiation of the program at the study center were available for analysis. The dataset therefore includes only successfully transplanted patients and does not capture censored cases, such as patients remaining on the waiting list, those removed from the program, or those who did not ultimately receive a transplant. As a result, the analysis underestimate the true level of system congestion and waiting times, since longer‐duration and unresolved cases are not captured. Consequently, the findings are interpreted as reflecting the subset of patients who successfully exited the system rather than the full waiting‐list population. This implies that a latent backlog likely exists among unobserved incompatible donor–recipient pairs who remain on prolonged dialysis without successful matching, potentially following different arrival and waiting patterns that are not represented in the current model. These findings are probabilistic indicators of system behavior under uncertainty, rather than deterministic predictions, particularly given the limited sample size and resulting variability in parameter estimates. The interpretation is supported by complementary confidence interval, sensitivity, and regression analyses, all of which produced qualitatively consistent findings despite the uncertainty associated with the available data. These analyses provide evidence consistent with compatibility‐constrained donor–recipient matching being a major operational contributor to prolonged waiting times. Future work incorporating unserved pairs and extended datasets would further refine system‐level estimates and reinforce these insights. Including data on these unmatched pairs would enrich the mathematical model, potentially revealing longer tails in wait‐time distributions. Systematic learning from such limitations aligns with LHS principles, where each cycle of data collection, analysis, and reflection informs more robust models and future decision‐making.

To prevent patients from being further victimized, it is imperative for the government and healthcare policymakers of the country to make a detailed national policy towards kidney transplantation and establish a centralized registry of renal patients awaiting transplants, along with all types of available donors with their biological typing of kidneys. This can help manage logistic scheduling and planning of adequate transplants. A centralized renal transplant registry be supported by standardized data acquisition and interoperability measures for demographic, biological typing, and clinical status variables to enable seamless data exchange between dialysis units, transplant centers, and policy‐making bodies. Real‐time analytics and decision support integrating queue management algorithms into electronic health records can trigger alerts when waiting‐list congestion exceeds safe thresholds, exemplifying LHS feedback loops that proactively improve operational decisions. Likewise, a clear policy around data‐sharing protocols to maintain confidentiality while enabling multi‐center research and policy evaluation needs to be implemented with regular upskilling of health‐information managers, clinicians, and allied staff in registry maintenance, data quality assurance, and the interpretation of queueing‐model outputs for operational decision‐making. Such measures directly support proactive system management and practical guidance for improving healthcare delivery, which is consistent with the findings of this study. This kind of continuous data use, feedback, and adaptive policy action illustrates the LHS cycle, where practice informs evidence and evidence directly improves practice in a dynamic loop.

Currently, Nepal only has one transplant center with a PKE, which indicates that patients often face long waits and limited options, where many get victimized in the queue itself. Expanding regional PKE units, equipped with surgical teams, laboratory support, and data‐sharing infrastructure, allows operational learning across centers and reduces the burden on the primary facility. By equipping regional centers with the necessary surgical teams, laboratory support, and data‐sharing infrastructure, the burden on the primary facility can be reduced. Once multiple centers are operational, a network linking nephrology, surgery, immunology, and health information departments can harmonize referral pathways, match donor‐recipient pairs more efficiently, and balance caseloads.

Such a coordinated network exemplifies an LHS approach, where continuous monitoring and iterative adjustments improve system performance and patient outcomes. The numerical analyses performed in this study provide evidence consistent with biological compatibility constraints in donor–recipient matching being a major operational contributor to persistent congestion and prolonged waiting times of the M/M/1 model. This highlights the critical need for policy, registry, and operational interventions aimed specifically at improving compatibility matching, rather than only increasing service capacity. In addition, the findings support actionable guidance for healthcare administrators to anticipate congestion, adjust resource allocation proactively, and implement targeted interventions based on real‐time data feedback. Future analysis can be generalized to more sophisticated mathematical model like the M/M/n model incorporating multiple servers to evaluate how adding transplant centers and working on co‐ordination among them impacts wait times, utilization, and overall system performance. While this study focuses on PKE data, future work could extend the analysis to conventional closed‐donor transplant queues, applying the same Learning Health System framework to evaluate system performance, assess operational constraints, and inform iterative service improvements. Ultimately, this evidence‐informed, systems‐level framework provides a foundation for developing a self‐improving kidney transplant system in Nepal by supporting continuous monitoring, adaptive decision‐making, and data‐driven policy refinement, with the potential to improve equity of access, reduce waiting times, and strengthen the resilience of transplant service delivery.

## Author Contributions

**Samrajya Raj Acharya:** conceptualization, methodology, investigation, visualization, formal analysis, software, validation, resources, data curation, writing – original draft, review and editing. **Sushil Ghimire:** methodology, principal supervision, project administrator, writing – review and editing. **Ram Prasad Ghimire:** supervision.

## Funding

The authors have nothing to report.

## Disclosure

This study is a component of the health system‐based application segment of Samrajya Raj Acharya’s final academic dissertation [MATH 499, 6 credits], for the Bachelor of Science in Computational Mathematics at the Department of Mathematics, School of Science, Kathmandu University, in 2024 A.D. The work was conducted under the principal supervision of Assistant Professor Sushil Ghimire, PhD, and the mentorship of Professor Ram Prasad Ghimire, PhD. This study represents a pioneering research contribution from Nepal in the field of Transplantation Mathematics.

## Ethics Statement

The anonymized data and the institutional clearence to publish the research findings used in this study were obtained from the Government of Nepal, Ministry of Health and Population, Shahid Dharma Bhakta National Transplant Center, in accordance with institutional data access protocols and upon payment of the applicable data release fees.

## Conflicts of Interest

The authors declare no conflicts of interest.

## Data Availability

The data sets generated and analyzed during the current investigation are available upon reasonable request from the author.

## Endnotes

^1^
The years mentioned in this study are based on the Bikram Sambat (B.S.) calendar, which is officially used in Nepal. This calendar runs approximately 56.7 years ahead of the Gregorian calendar. For reference, the period from 2069 B.S. to 2080 B.S. corresponds to the years 2012/2013 A.D. to 2023/2024 A.D.
